# Applications of Neuroimaging to Disease-Modification Trials in Alzheimer’s Disease

**DOI:** 10.3233/BEN-2009-0241

**Published:** 2009-10-21

**Authors:** Adam S. Fleisher, Michael Donohue, Kewei Chen, James B. Brewer, Paul S. Aisen, the Alzheimer’s Disease Neuroimaging Initiative

**Affiliations:** ^1^Department of NeurosciencesUniversity of CaliforniaSan DiegoUSA; ^2^Department of RadiologyUniversity of CaliforniaSan DiegoUSA; ^3^Division of Biostatistics and BioinformaticsUniversity of CaliforniaSan DiegoUSA; ^4^Banner Alzheimer's InstitutePhoenixAZUSA

## Abstract

Critical to development of new therapies for Alzheimer’s disease (AD) is the ability to detect clinical or pathological change over time. Clinical outcome measures typically used in therapeutic trials have unfortunately proven to be relatively variable and somewhat insensitive to change in this slowly progressive disease. For this reason, development of surrogate biomarkers that identify significant disease-associated brain changes are necessary to expedite treatment development in AD. Since AD pathology is present in the brain many years prior to clinical manifestation, ideally we want to develop biomarkers of disease that identify abnormal brain structure or function even prior to cognitive decline. Magnetic resonance imaging, fluorodeoxyglucose positron emission tomography, new amyloid imaging techniques, and spinal fluid markers of AD all have great potential to provide surrogate endpoint measures for AD pathology. The Alzheimer’s disease neuroimaging initiative (ADNI) was developed for the distinct purpose of evaluating surrogate biomarkers for drug development in AD. Recent evidence from ADNI demonstrates that imaging may provide more sensitive, and earlier, measures of disease progression than traditional clinical measures for powering clinical drug trials in Alzheimer's disease. This review discusses recently presented data from the ADNI dataset, and the importance of imaging in the future of drug development in AD.

## 1. Introduction: A need for biomarkers of disease in drug development

In the last two decades Alzheimer's disease (AD) research has made many leaps forward towards the goal of finding a cure for AD. In particular, beta amyloid pathology and neurofibrillary tangles have been identified as highly associated with the clinical presentation of AD [1,11]. And, an early onset AD clinical syndrome and pathology have been linked to autosomal dominant kindreds with mutations in genes responsible for amyloid precursor protein metabolism. Another important discovery was of the apolipoprotein epsilon 4 allele (APOE4), which is currently the most potent known genetic risk for late onset AD [3]. Current drug development strategies primarily focus on treating AD after cognitive symptoms have already begun, after meeting clinical dementia criteria, or in clinical prodromal states such as mild cognitive impairment (MCI) [30,42]. However, unlike most diseases that present shortly after the onset of underlying pathology, we now realize that AD pathology begins many years (possibly decades) prior to clinical manifestations [7,

[1]Data used in the preparation of this article were obtained from the Alzheimer's Disease Neuroimaging Initiative (ADNI) database (www.loni.ucla.edu\ADNI). As such, the investigators within the ADNI contributed to the design and implementation of ADNI and/or provided data but did not participate in analysis or writing of this report. ADNI investigators include (complete listing available at http://www.loni.ucla.edu/ADNI/Data/ADNI_Authorship_List.pdf).

ISSN 0953-4180/09/$17.00 © 2009 – IOS Press and the authors. All rights reserved

24]. And, once functional impairment occurs, it may be difficult to derail the neurodegenerative process, making it unlikely that we will be able to return individuals to their pre-morbid cognitive state. Therefore, a cure for Alzheimer's disease is most likely to be found by detecting Alzheimer's disease pathology at its earliest clinical stage, or potentially even before clinical manifestations. For this, we need more sensitive measures of clinical symptoms or underlying pathology to power clinical trials.

Efforts to demonstrate disease-modifying effects in AD have been frustratingly unsuccessful. While many plausible targets and candidate agents have been developed, there have been no successful efficacy trials (though several large trials are now in progress). In particular, failures of three putative anti-amyloid therapies in AD, Flurizan, Alzemed and AN1792 underscored the need to improve abilities to detect disease modifying effects of putative AD therapies [38]. The failure of completed trials to demonstrate benefits may reflect low potency of the interventions, but it is quite likely that methodological difficulties have contributed. Indeed, there is a consensus that it is essential to improve trial design to facilitate the development of the next generation of AD therapies. Neuroimaging biomarkers will be an important component of optimal trial design.

All of the AD drugs currently approved provide modest symptomatic benefits that last twelve to eighteen months, on average [43]. There is no evidence that these drugs modify underlying disease pathology or curtail the ultimate progression of disease and clinical decline. Symptomatic improvement in cognition and function or global status can be demonstrated in 3–6 month trials; maximal separation of treatment and placebo groups on primary outcome measures occurs in this time frame [43]. Because these effects can be seen in a relatively short period of time, it has been feasible to demonstrate benefits and select optimal doses in Phase II trials that have been predictive of success in pivotal Phase III studies of symptomatic therapies.

Disease-modifying drugs, however, will not necessarily show any short-term symptomatic benefits. These interventions, attacking targets along the amyloid or neurofibrillary tangle cascades or otherwise providing neuroprotection, aim to slow the rate of neurdegeneration causing cognitive and clinical decline. Unlike symptomatic treatments, they are not particularly aimed at "boosting" short term clinical performance. Since there is often little or no placebo group decline observed in mild AD trials of 6 months or less, long trials are necessary to see an efficacy signal. Indeed, the European Medicines Agency guidelines indicate that trials of at least 18 months are required to document disease-modifying effects. Dose-finding efficacy trials are therefore not feasible in a standard-type 3-6 month Phase II program.

This difficulty is compounded by the growing concern that the mild AD population is too advanced in terms of extent of amyloid and tangle neuropathology to show substantial benefits with disease-modifying interventions [38]. Pathology likely precedes dementia onset by a decade or longer, with dementia onset representing a late stage along the neurobiological pathway [8,24]. For this reason, to optimize the impact of disease-modifying treatments, they must be initiated at the earliest possible stage of disease. But at an early, pre-dementia stage of disease, decline rates on standard cognitive and clinical measures are quite gradual, reducing the power of trials to demonstrate effects. Clinician-based assessments or cognitive measures such as the Alzheimer's Disease Assessment Scale (ADAS-Cog) [36] and the Clinical Dementia Rating Scale (CDR) [26], are commonly used to evaluate treatment efficacy in such clinical trials, though both are limited in their sensitivity to detect change over time and require the use of large sample sizes and extended observation times [12,17,27]. To improve detection of significant treatment effects in early clinical or pre-clinical disease, it is therefore necessary to develop more sensitive surrogate endpoint measurer of disease progression.

Neuroimaging biomarkers offer some solutions to these difficulties. Longitudinal studies such as the Alzheimer's Disease Neuroimaging Initiative (ADNI) have demonstrated the power of neuroimaging to reflect AD neurobiology [14,21,25,28]. It is now feasible to utilize neuroimaging to advance trial design in three ways: to select early-stage subjects for trials, to provide covariates to reduce unexplained variance in cognitive and clinical progression and thereby increase trial power, and as surrogate outcome measures.

The studies reviewed here represent analyses of imaging and spinal fluid data collected in the ADNI, and obtained from the publicly available ADNI database (http://www.loni.ucla.edu\ADNI). The ADNI was launched in 2003 by the National Institute on Aging (NIA), the National Institute of Biomedical Imaging and Bioengineering (NIBIB), the Food and Drug Administration (FDA), private pharmaceutical companies and non-profit organizations, as a $60 million, 5-year public-private partnership. The primary goal of ADNI has been to test whether serial magnetic resonance imaging (MRI), positron emission tomography (PET), other biological markers, and clinical and neuropsychological assessment can be combined to measure the progression of mild cognitive impairment (MCI) and early Alzheimer's disease (AD). Determination of sensitive and specific markers of very early AD progression is intended to aid researchers and clinicians to develop new treatments and monitor their effectiveness, as well as lessen the time and cost of clinical trials. The Principle Investigator of this initiative is Michael W. Weiner, M.D., VA Medical Center and University of California – San Francisco. ADNI is the result of efforts of many co-investigators from a broad range of academic institutions and private corporations, and subjects have been recruited from over 50 sites across the US and Canada. The initial goal of ADNI was to recruit 800 adults, ages 55 to 90, to participate in the research – approximately 200 cognitively normal older individuals to be followed for 3 years, 400 people with MCI to be followed for 3 years, and 200 people with early AD to be followed for 2 years. For up-to-date information see www.adni-info.org.

## 2. Neuroimaging and subject selection

Attempts to study disease-modifying interventions at a clinical stage earlier than mild dementia have generally focused on MCI [30,42]. The population defined by commonly used guidelines for MCI is somewhat heterogeneous; it includes individuals who may not show substantial decline during the years following diagnosis. Further, rate of progression to dementia has varied substantially among various MCI studies [32]. Neuroimaging and cerebrospinal biomarker studies such as ADNI and those at Washington University and the University of Guthenburg have clarified the neurobiological events underlying AD and its prodromal stages. Evidence suggests that amyloid deposition occurs years before dementia onset, and after a lag period without significant symptoms, amnestic MCI develops with gradual progression to AD [14]. It therefore seems reasonable to select subjects with the cognitive criteria of MCI plus biomarker evidence of amyloid as an ideal population for pre-dementia trials of disease-modifying drugs, particularly anti-amyloid drugs [6]. Cerebral spinal fluid (CSF) biomarkers and recent amyloid imaging techniques may provide an opportunity to enrich subject selection in nearly treatment trials.

ADNI data provides an opportunity to test this idea [13,28]. Subjects with brain amyloid can be identified either by amyloid PET imaging [19,29] (either with $^{11}$C-PIB [18] or the newer $^{18}$F amyloid ligands) or by the presence of a low CSF amyloid beta 42 ($A\beta_{42}$) level [39]. Since more ADNI subjects have had CSF collection than amyloid imaging, our group has explored the value of subject selection based on levels of CSF amyloid. We find a substantial gain in statistical power, as expected. For an MCI study with a two year treatment period aiming to demonstrate a 40% slowing of disease progression as indicated by decline on continuous measures of cognition or clinical stage, selection of subjects with CSF amyloid level cut-offs reduces the necessary sample size by 35%. Similarly, such selection reduces sample size of an MCI study using a time-to-dementia analysis, yielding a 30% reduction in sample size [4,5] (http://www.adni-info.org/images/stories/SteeringCommittee2009/03-1_adni%20early%20ad%20trial.pdf). Likewise, it may also be possible to preselect subjects for clinical trials based on degree of amyloid present on amyloid PET imaging. Evidence suggests from ADNI that PiB positivity is associated with worsened episodic memory and hippocampal volumes in MCI subjects [25], and may indicate higher rates of conversion to AD. Utilizing these biomarkers to reduce sample sizes needed to demonstrate drug effects on clinical measure will make studies of treatment effects in MCI readily feasible. Further, it is obviously appropriate to limit subjects in anti-amyloid treatment trials to those with evidence of amyloid accumulation.

## 3. Neuroimaging and covariates for analysis of standard outcome measures

Amyloid imaging and CSF $A\beta$ measurements identify subjects with amyloid dysregulation and accumulation, but do not show significant longitudinal change in subjects with MCI or AD [7,14]. On the other hand, volumetric MRI measures, including hippocampal volume, whole brain volume, ventricular enlargement and regional cortical thickness measures, show predictable longitudinal decline that tracks clinical progression [14–16,23]. Likewise, measures of flurodeoxyglucose (FDG) PET, a measure of glucose metabolisms also reflect stage of clinical disease, as demonstrated in the ADNI cohort [22]. This provides an accurate and objective measure of disease stage, which in turn predicts subsequent cognitive and clinical decline. It is therefore useful to utilize volumetric MRI measures or FDG PET as covariates in the analysis of longitudinal data from MCI and AD trials [22, 44]. For example, we assessed various ADNI imaging and biomarker measures as covariates to identify their influence on required sample sizes in a 24 month clinical trial of MCI, utilizing decline on the ADAS-cog$_{12}$ or the Clinical Dementia Rating Scale – sum of boxes (CDR-SB) as efficary endpoint measures. Using linear random effect modeling we assumed a 30% effect size with a power of 80% and a 2-sided alpha equal to 0.05. If just utilizing decline on the ADAS-cog$_{12}$ as an endpoint measure, it would require 593 subjects to detect a 30% effect size. If cortical thickness in the temporal lobe is added as a covariate to this model, this number can be reduced by 10% to 534 (http://www.adni-info.org/images/stories/SteeringCommittee2009/03-1_adni%20early%20ad%20trial.pdf). Further evaluation of how various imaging techniques can be used as covariates in endpoint modeling may prove to enhance our ability to detect drug effects and reduce necessary sample sizes and study lengths.

## 4. Neuroimaging for trial endpoints

A surrogate endpoint is a marker of underlying disease that reflects clinical and/or pathological disease processes with a high degree of specificity and sensitivity. For a biomarker to be accepted as a surrogate endpoint in a clinical drug trial it must (1) be correlated with the clinical endpoint; and (2) fully capture the net effect of the intervention on the clinical efficacy endpoint [10, 31]. The regulatory agencies require demonstration of benefits on two co-primary outcome measures for approval of treatments for AD, a broad cognitive measure plus an assessment of global clinical status or function. As noted above, it requires a large trial to demonstrate such effects in AD and especially in pre-dementia populations. However, the effect of an intervention on synaptic function (presumably underlying the cognitive and clinical changes) can be assessed using FDG-PET, providing a potential method for exploring benefit in smaller studies. This has been utilized in a small Phase 1 study of a neuroprotective intervention in mild to moderate AD [40], and may have wider utility across the spectrum of disease [33–35]. Reiman et al, demonstrated that the ADNI FDG-PET dataset can be utilized to improve power to detect disease progression and disease modifying treatment effects when used as a primary treatment effect endpoint (presented at the 2009 ADNI meeting, April 27th, Seattle, WA: http://www.adni-info.org/images/stories/SteeringCommittee2009/06_jagust.pdf). A training dataset consisting of baseline to 12 month follow-up FDG-PET scans from 27 AD subjects from the ADNI dataset was used to optimize settings for the detection of longitudinal FDG-PET signal decline. This was done by creating an empirically defined statistical region of interest (sROI) that identifies brain voxels whose mean change serves as a reliable index for decline in FDG PET signal over 12 months (Fig. 1). Utilizing a second testing dataset (29 AD), mean change in this pre-defined sROI was used as a primary outcome measure for a power analysis to determine detectable change over 12 months in ADNI AD subjects. This power analysis assumed a treatment effect size of 25%, 80% power with an alpha=0.05. To identify significant changes in FDG PET signal consistent with those seen in AD, only 61 AD patients would be required in a 12 month study. This is compared to 673 subjects for similar effects to be recognized with decline in CDR-SB, 612 with the ADAS-cog$_{11}$, or 493 for change in Mini Mental State Exam Scores in this same cohort.

Both MRI volumetric endpoints and FDG PET measures may be useful for improving power in clinical trials or even used as primary outcome measures. MRI is more specific for establishing a disease-modifying treatment effect. FDG-PET, on the other hand, can show reversible symptomatic effects as well as disease-modifying effects. Analysis of ADNI data demonstrates that MRI data is also substantially more powerful than cognitive endpoints, providing a feasible method for exploring effects in Phase II trials, and for establishing disease-modifying effects in Phase III studies.

MRI volumetric measures have been well established to distinguish Alzheimer's disease and MCI from nondemented individuals, as well as predict future cognitive decline [14,16,37,41]. Recently, ADNI data has provided an opportunity to assess potential uses of volumetric MRI data as primary outcome measures in multicenter randomized clinical trials, and determine power for detecting change over time. In an ADNI volumetric MRI study by our group [20], we characterized 12-month whole brain atrophy rates in 34 probable AD patients (pAD), 75 amnestic MCI patients, and 38 elderly NC using Iterative Principal Component Analysis (IPCA). Using IPCA [2], whole brain atrophy annual percent change was determined to be $0.60 \pm 0.23\%$ in pAD patients, $0.37 \pm 0.28\%$ in MCI patients, and $0.28 \pm 0.24\%$ in NC (ANOVA $P = 1e\text{-}6$, linear trend

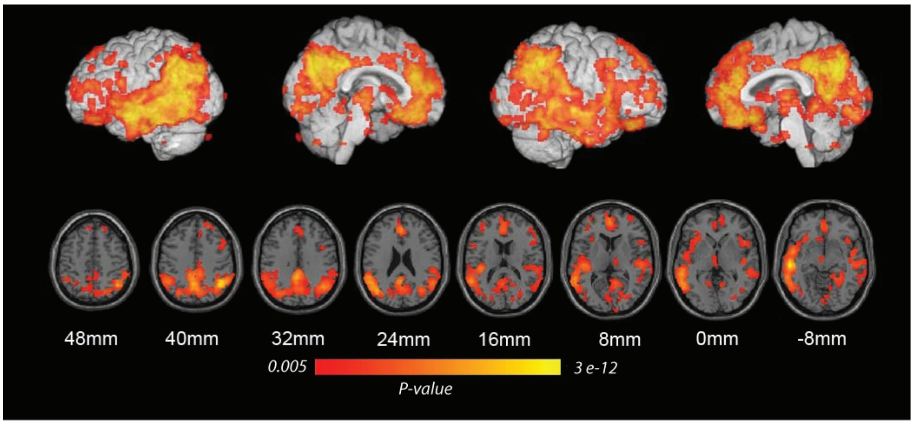

Fig. 1. Statistical pattern of FDG PET change over 12 months in AD sample. Statistical region of interest representing 12 month FDG PET signal change in a training set of 27 subjects from the ADNI dataset. This pattern of AD-like change can be used as an outcome measure to identify effects of putative treatments for AD in randomized clinical trials.

P = 7e-7). For pAD, power estimates determined the need for 37 patients using this measure of brain atrophy, compared to 474 using the ADAS-COG to detect a 25% AD-slowing treatment effect with 80% power and two-tailed P = 0.05 in twelve-month multi-center randomized controlled trials (RCTs).

Thompson et al. recently used the ADNI MRI dataset to demonstrate that tensor based morphometry (TBM), a sensitive technique that measures volumetric changes in brain structures, can be used as an endpoint measure to significantly improve effect sizes and reduce sample sizes in clinical trials evaluating disease modifying effects [12]. Applying the sROI approach introduced by Reiman et al (Fig. 1), this study exemplifies the areas of change over time in AD ($n = 104$), MCI ($n = 254$), and normal controls (157). Detection power was greatly enhanced by summarizing changes in a statistically-defined region-of-interest derived from an independent training sample of 22 AD patients. In power analyses, the best method required only 48 AD and 88 MCI subjects to give 80% power to detect a 25% reduction in the mean annual change using a two-sided test (at $\alpha =$ 0.05). This represents a dramatic sample size reduction compared to using clinical scores as outcome measures (619 AD/6797 MCI for the ADAS-Cog, and 408 AD/796 MCI for the Clinical Dementia Rating sum-of-boxes scores). Table 1 compares sample size estimates using FDG-PET, TBM-MRI, whole-brain atrophy and clinical measures as primary endpoint measures for a hypothetical clinical drug trial in AD, compiled from the studies presented here. It is not clear which imaging technique provides the most power to detect AD-associated brain changes over time. More studies are needed. However, it is clear that using imaging as primary endpoints provides advantages compared to using standard cognitive endpoint measures in clinical trials of AD.

Table 1
Treatment trial sample size estimates using FDG PET and MRI morphometry compared to clinical measures as primary endpoints for AD clinical trials

| | Sample size estimates |
|---|---|
| sROI-FDG-PET | 61 |
| TBM-MRI* | 48 |
| Whole brain atrophy: IPCA -MRI | 37 |
| CDR-SB | 673 |
| ADAS-Cog | 612 |
| MMSE | 493 |

Estimated sample size per treatment arm in a treatment trial for Alzheimer's disease needed to detect a 25% reduction in mean annual change with a two-sided test and $\alpha = 0.05$ at 80% power (pooled data, see text). *Adapted from [12].

Many investigators suspect that the ideal population for disease-modifying interventions is the group with amyloid accumulation but no significant symptomatology. Cognitive and clinical measures are not useful in assessing drug effects in this "pre-symptomatic" group. But based on preliminary evidence from ADNI, such subjects do show increased atrophy rates, as well as changes in FDG PET and amyloid imaging, so it may be possible to demonstrate drug effects using these imaging measures. It must be emphasized though that until treatment effects on such measures are shown to correlate with cognitive and clinical measures, they will not be considered primary (ie, surrogate) endpoints for pivotal trials. Nevertheless, as of now, these types of correlations are most promising for MRI volumetrics and FDG PET [9,14,21,23]. Amyloid PET imaging also holds great promise for measuring therapeutic effects on cortical amyloid load for treatments that directly target amyloid pathology. Although the ADNI is not designed to assess how well biomarkers capture intervention effects, it is well positioned to give researches opportunities to develop surrogate biomarkers for this purpose.

## 5. Conclusion

Improvements to clinical trial methodology will facilitate the development of disease-modifying treatments for AD. In particular, neuroimaging modalities offer a method for subject selection and characterization that can greatly improve statistical power. This is critically important for trials in AD dementia, and even more crucial for trials conducted in mildly impaired, pre-dementia populations with very gradual decline rates. Analysis of the publically available ADNI data has confirmed the utility of imaging biomarkers for trial design; such analyses provide a framework for the incorporation of standardized neuroimaging measures into multicenter trials. Adoption of these methods by drug development programs can link treatment effect on neuroimaging measures to standard cognitive and clinical outcomes, the essential step toward the goal of establishing neuroimaging biomarkers as surrogate primary endpoints in AD clinical trials to facilitate the development of treatments for pre-symptomatic individuals.

## Acknowledgments

ADNI data presented in this review were funded through the following sources: The Foundation for the National Institutes of Health (www.fnih.org) coordinates the private sector participation of the $60 million ADNI public-private partnership that was begun by the National Institute on Aging (NIA) and supported by the National Institutes of Health. To date, more than $27 million has been provided to the Foundation for NIH by Abbott, AstraZeneca AB, Bayer Schering Pharma AG, Bristol-Myers Squibb, Eisai Global Clinical Development, Elan Corporation, Genentech, GE Healthcare, GlaxoSmithKline, Innogenetics, Johnson & Johnson, Eli Lilly and Co., Merck & Co., Inc., Novartis AG, Pfizer Inc., F. Hoffmann-La Roche, Schering-Plough, Synarc Inc., and Wyeth, as well as non-profit partners the Alzheimer's Association and the Institute for the Study of Aging.
